# Multi-Territorial Ischemic Stroke Following Russell’s Viper Envenomation: A Case Report

**DOI:** 10.7759/cureus.87988

**Published:** 2025-07-15

**Authors:** Pooja Yadav, Manoj Kumar, Vijay Sundarsingh, Akshata Charlotte, Monish Thomas

**Affiliations:** 1 Anesthesia, Safdarjung Hospital, New Delhi, IND; 2 Anesthesiology, Father Muller Medical College, Mangalore, IND; 3 Critical Care Medicine, Father Muller Medical College, Mangalore, IND; 4 Ophthalmology, Father Muller Medical College, Mangalore, IND

**Keywords:** ischemic cerebrovascular disease, multi-territorial infarct, ptosis, russell's viper, snake envenomation, venom-induced consumptive coagulopathy

## Abstract

Snake envenomation is a major health concern in tropical countries. Russell’s viper, one of the "big four" venomous snakes in India, is known for its hemotoxic venom that commonly causes coagulopathy, spontaneous bleeding, and local tissue damage. However, ischemic strokes following viper envenomation are rare and underreported. We describe a case of a 52-year-old healthy male who sustained Russell’s viper bite, and he presented with profuse sweating, altered sensorium, and respiratory distress within hours of the bite and required mechanical ventilation and anti-snake venom (ASV) therapy. Despite timely ASV administration, the patient developed progressive neurological symptoms, including ptosis. MRI brain revealed multi-territorial acute infarcts involving the left parietal-temporal-occipital cortex, pons, and cerebellum. Stroke evaluation ruled out common etiologies, such as atherosclerosis, cardioembolism, and vasculitis. Coagulation studies revealed evidence of venom-induced consumption coagulopathy (VICC), suggesting that the procoagulant components of viper venom led to widespread thrombosis. With supportive care and antiplatelet therapy, the patient gradually improved and was discharged with residual aphasia, requiring rehabilitation. This case highlights an unusual but serious complication of Russell’s viper envenomation, a multi-territorial acute ischemic stroke that was likely mediated by a venom-induced prothrombotic state. Clinicians should maintain a high index of suspicion for thrombotic events in patients with altered mental status following a viper bite, even in the absence of traditional stroke risk factors.

## Introduction

Snakebite envenomation remains a major but under-recognized global health challenge, affecting an estimated 2.7 million people annually [1]. In 2017, the World Health Organization officially designated it as a Category A Neglected Tropical Disease, highlighting the need for greater awareness, research, and access to treatment [2]. The burden of snakebite is particularly high in tropical and subtropical regions, especially Southeast Asia and sub-Saharan Africa. India carries the highest burden globally, with over 250,000 snakebite cases reported annually and an estimated 1.2 million deaths between 2000 and 2019 [3]. Snake envenomation can cause acute medical emergencies involving local tissue damage, spontaneous hemorrhage, neuromuscular paralysis, shock, acute kidney injury, and cerebrovascular events [4].

Among venomous snakes in India, the Russell’s viper (*Daboia russelii*) is responsible for a significant number of envenomation-related fatalities. This snake’s venom is predominantly hemotoxic and is known to cause a range of complications [5]. Neurological manifestations, though less common than hemotoxic complications, may include neuromuscular paralysis and, in rare cases, cerebrovascular complications. Hemorrhagic stroke has been documented in the context of viper envenomation due to venom-induced coagulopathy. In contrast, ischemic strokes are considered rare and underreported complications. The mechanisms proposed include venom-induced vascular endothelial damage, hypercoagulable states, thrombotic microangiopathy, or cardioembolism secondary to venom effects.

This report describes an unusual case of multi-territorial acute ischemic stroke following envenomation by a Russell’s viper. The case adds to the limited literature on arterial thrombotic events following hemotoxic snakebite and highlights the importance of recognizing ischemic complications in this setting. Early detection and neuroimaging are crucial for diagnosis, especially in patients presenting with altered sensorium or focal neurological deficits post-envenomation.

## Case presentation

A 52-year-old previously healthy Indian male sustained a snakebite to his left leg while gardening. The offending snake was later identified as a Russell’s viper (*Daboia russelii*). Within minutes of the bite, the patient experienced profuse sweating and collapsed. He was immediately rushed to a nearby hospital, where he was noted to have altered sensorium and respiratory distress. Bedside whole blood clotting time (WBCT) was abnormal, raising suspicion of venom-induced coagulopathy. He was intubated for airway protection and received an initial dose of 10 vials of polyvalent anti-snake venom (ASV).

Due to his worsening neurological status and requirement for mechanical ventilation, the patient was transferred to our tertiary care facility for further management. On arrival, he was restless, with a Glasgow Coma Scale (GCS) score of E3VTM6. Pupillary reflexes were preserved, and bilateral plantar reflexes were absent. Vital signs included a heart rate of 110 beats/min, blood pressure of 150/70 mmHg, and respiratory rate of 20 breaths/min. The electrocardiogram showed sinus rhythm. A chest radiograph was performed, which showed clear lung fields with no signs of consolidation, effusion, or other acute pulmonary pathology. Additionally, arterial blood gas (ABG) analysis was within normal limits, indicating adequate oxygenation and ventilation. The left foot showed localized swelling and erythema with continuous bleeding from the bite site. He also had active nasal and oral bleeding and purpuric spots over his chest.

Bedside WBCT at our center remained abnormal, and another 10 vials of ASV were administered. The 10 vials of ASV were diluted in 500 mL of 5% dextrose (D5%) and administered as an intravenous infusion over 1 hour. He was started on intravenous cefotaxime, and tetanus toxoid was given. Initial laboratory evaluation revealed marked leukocytosis, elevated D-dimer levels, positive fibrin degradation products, and low fibrinogen, consistent with venom-induced consumptive coagulopathy (Table 1). Urinalysis revealed hematuria with 15-20 RBCs/high power field. He was transfused with four units of fresh frozen plasma (FFP) and 10 units of cryoprecipitate. Despite this, his WBCT remained abnormal after 8 hours, prompting a third dose of 10 vials of ASV. By 24 hours post-admission, his coagulation profile began to normalize, and the bleeding subsided.

**Table 1:** Laboratory investigations during the initial phase of ICU stay. Peripheral smear showed thrombocytopenia and no schistocytes. WBCT: whole blood clotting time; INR: international normalized ratio; APTT: activated partial thromboplastin time; FDP: fibrin degradation products

Parameters	At admission	24 hours	48 hours	Reference range
Fibrinogen	34 mg/dL	152 mg/dL	482 mg/dL	200-400 mg/dL
WBCT	>20 min	Clotted <20 min	Clotted <20 min	Clots within 20 min
INR	2.48	1.25	1.12	0.8-1.2
aPTT	76.1 s	21.3 s	20.8 s	25-35 s
Platelet count	168,000/µL	85,000/µL	91,000/µL	150,000-450,000/µL
Hemoglobin	14.4 g/dL	11.6 g/dL	11.9 g/dL	Male: 13.5-17.5 g/dL, female: 12-15.5 g/dL
Total leukocyte count	18,540/µL	10,490/µL	10,770/µL	4,000-11,000/µL
D-dimer	>35,200 ng/mL	NA	NA	<500 ng/mL
FDP	Positive (>10 µg/mL)	NA	NA	<10 µg/mL

**Figure 1:**
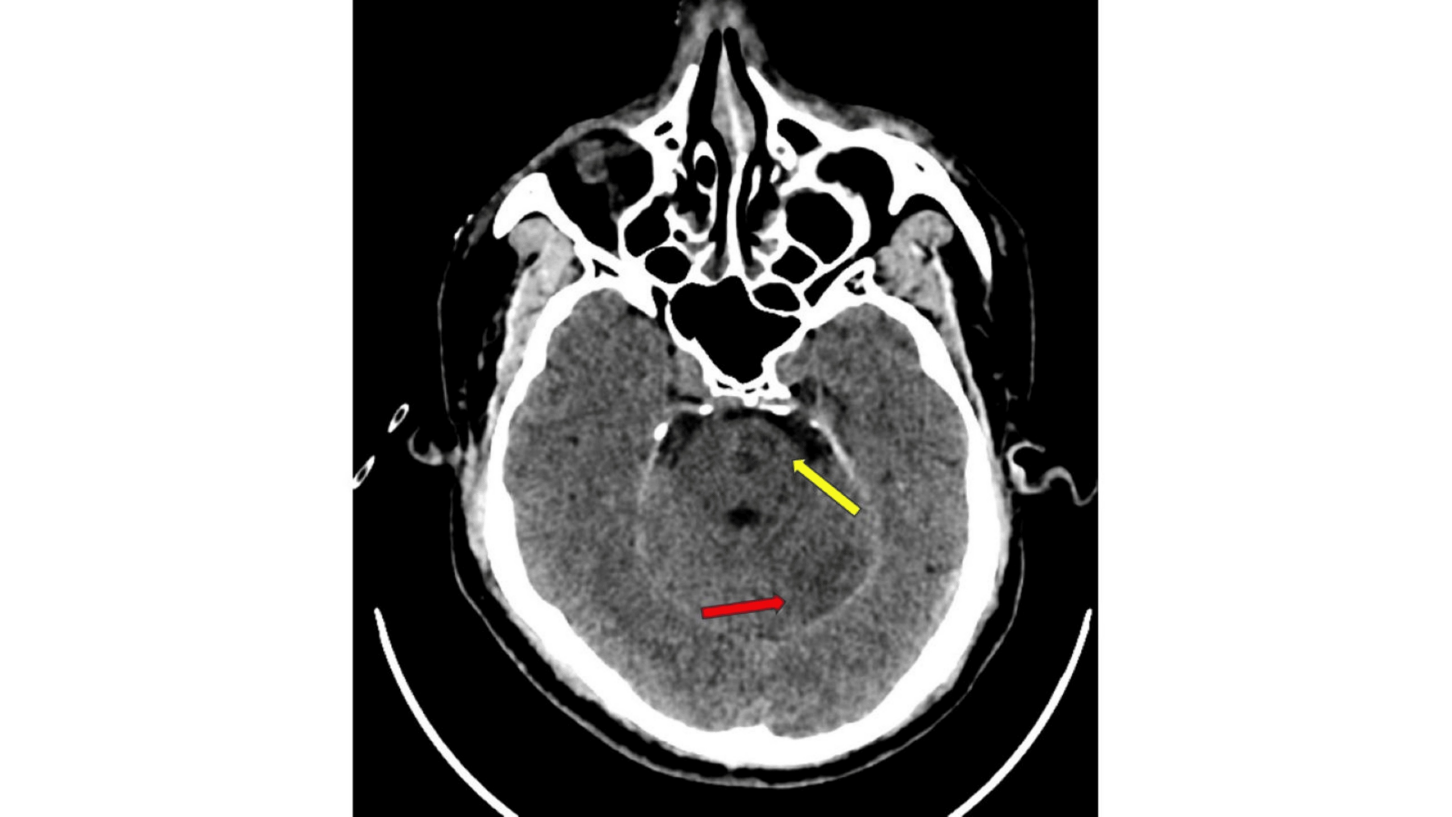
Non-contrast CT brain showing infarcts in the brain stem and cerebellum. The CT image shows acute infarcts in the left brainstem (pons - yellow arrow) and left cerebellar peduncle (red arrow), as indicated by the hypodense areas marked by the arrows.

By day two, as oral and nasal bleeding had ceased, sedation was withheld to reassess neurological function. The patient was awake but disoriented, with a GCS of E4VTM5. No limb weakness was observed. Notably, he developed ptosis and ophthalmoplegia in the left eye. A non-contrast computed tomography (NCCT) scan of the brain showed early, subtle hypodensities in the left brainstem and cerebellum suggestive of acute infarction (Figure 1). We administered aspirin at a loading dose of 325 mg along with clopidogrel 300 mg through a nasogastric tube after careful risk-benefit evaluation. A 2D echocardiogram showed no valvular pathology or intracardiac thrombus. Carotid and vertebral artery Doppler studies were unremarkable. His lipid profile and serum homocysteine were within normal limits. He was a lifelong non-smoker and consumed no alcohol.

**Figure 2:**
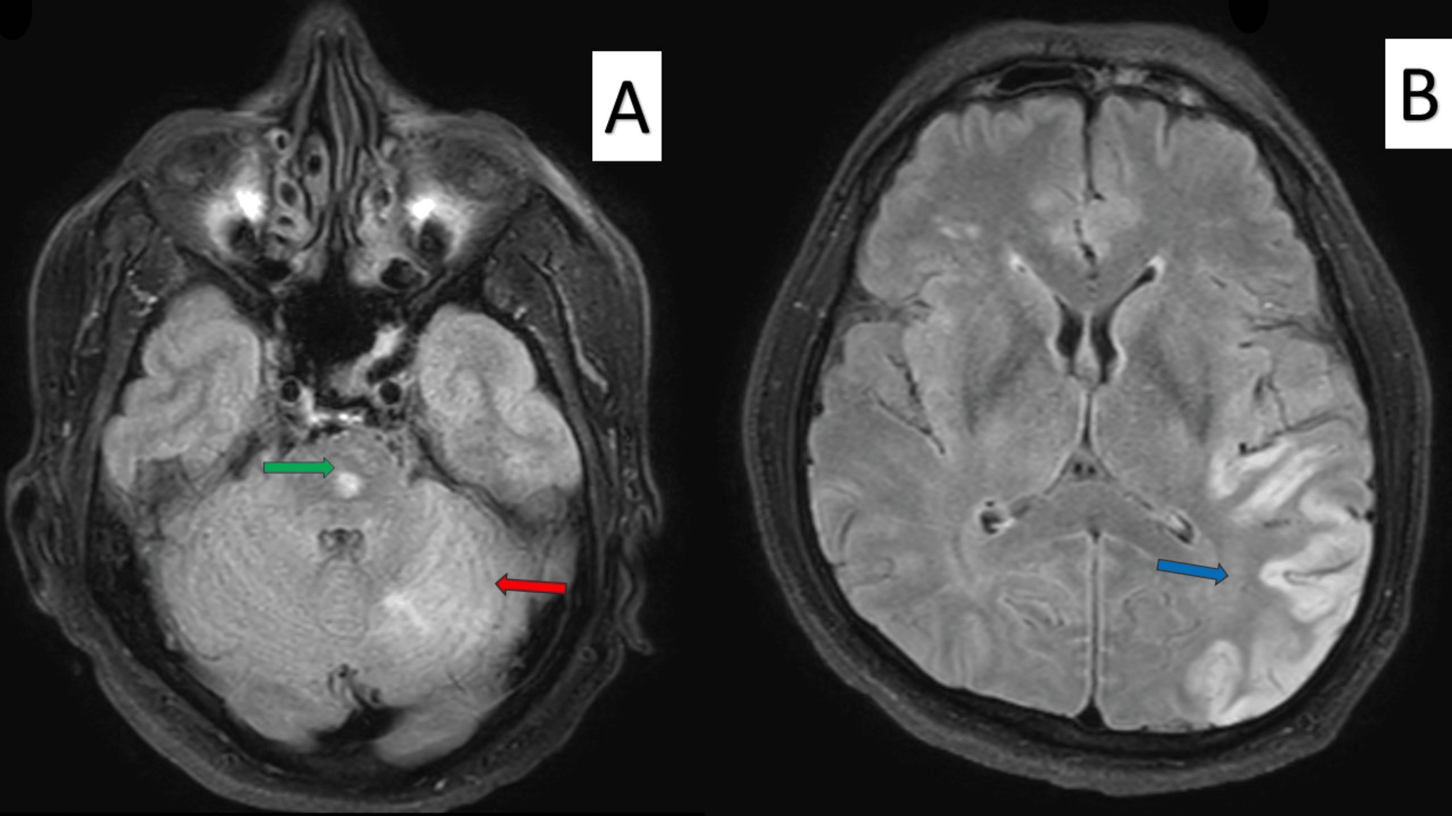
MRI brain showing multi-territorial infarcts in FLAIR sequence. (A) Acute infarcts in the left brain stem (green arrow) and cerebellum (red arrow). (B) Acute infarcts in the left parietal, temporal, and occipital lobes (blue arrow). FLAIR: fluid-attenuated inversion recovery

On day three, a spontaneous breathing trial was attempted but failed due to restlessness and agitation. The patient was diagnosed with hyperactive delirium, confirmed by a positive assessment using the Confusion Assessment Method for the Intensive Care Unit (CAM-ICU), and was managed with a combination of dexmedetomidine and haloperidol for symptom control. On day four, his GCS declined to E3VTM4, prompting further evaluation. Magnetic resonance imaging (MRI) of the brain revealed acute infarcts in multiple arterial territories, including the left parieto-temporo-occipital cortex, the left hemispheric pons, and the left cerebellar hemisphere. These areas appeared hyperintense on T2-fluid-attenuated inversion recovery (FLAIR) with restricted diffusion on diffusion-weighted imaging (DWI) sequences, confirming the diagnosis of multi-territorial acute ischemic stroke (Figures 2A, 2B).

**Figure 3:**
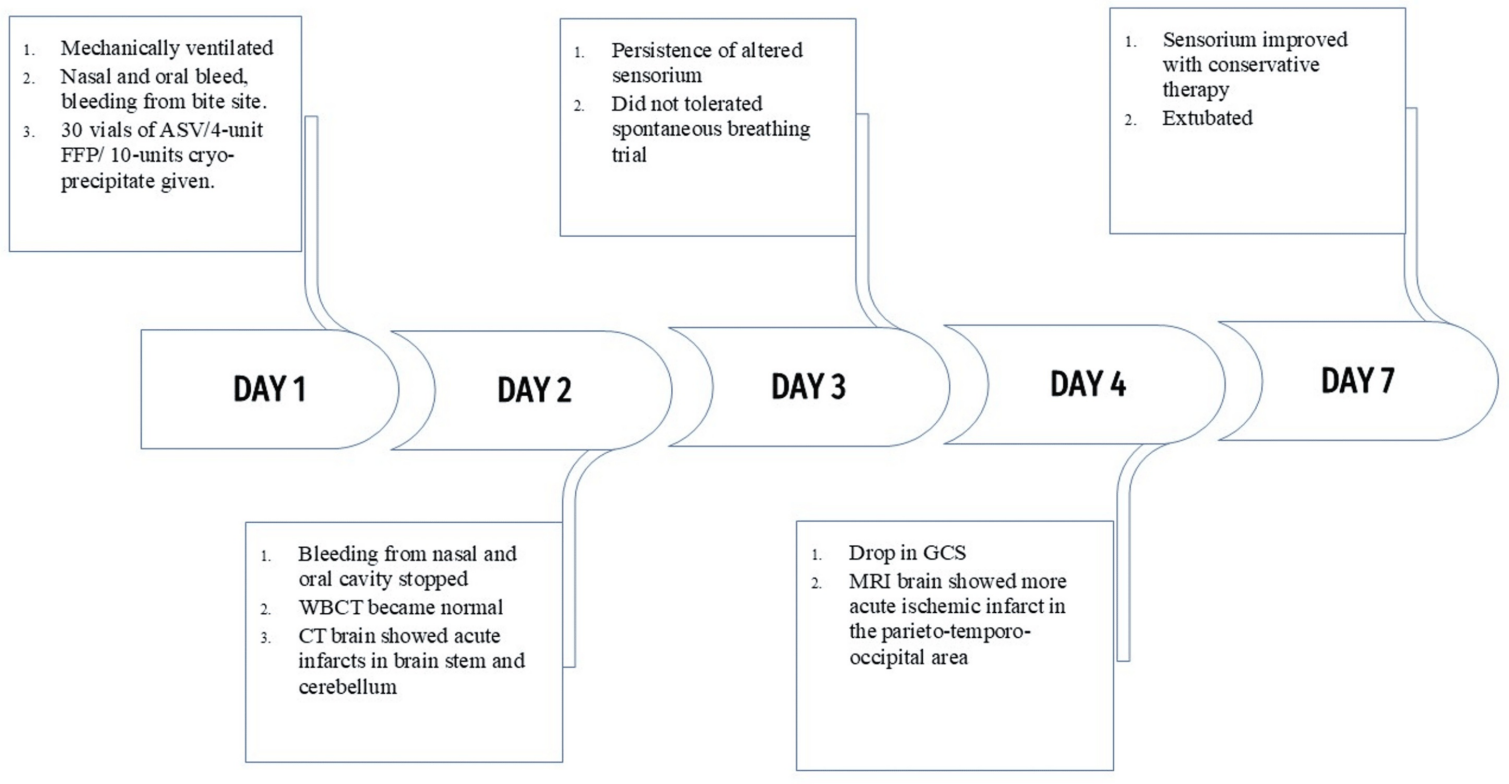
Timeline of events during hospital stay. ASV: anti-snake venom; FFP: fresh frozen plasma; WBCT: whole blood clotting time; GCS: Glasgow Coma Scale

Supportive care, close neurological monitoring, and dual antiplatelet therapy with tablets of aspirin and clopidogrel at 75 mg each were continued. The patient's sensorium gradually improved over the next few days. On day seven, he was successfully extubated. Post-extubation, he was noted to have aphasia, likely due to the cortical infarcts. A structured neuro-rehabilitation plan was initiated, including daily sessions with a speech-language pathologist and physiotherapist. The patient showed slow but steady functional improvement and was transferred to the ward for continued care. The timeline of clinical events from envenomation to recovery during hospitalization is illustrated in Figure 3.

## Discussion

Viper envenomation is typically associated with hemotoxic manifestations, including venom-induced consumption coagulopathy (VICC), bleeding diathesis, local tissue necrosis, and cellulitis. While hemorrhagic complications, such as intracranial hemorrhage, are well documented, acute ischemic infarctions remain exceedingly rare. Only a handful of case reports in the literature have described cerebral infarction following viper envenomation [6-9]. Nevertheless, neurological manifestations in such cases often indicate severe systemic toxicity.

VICC is recognized as the most significant systemic complication of viper bites globally. It results from the activation of the coagulation cascade by venom components, such as serine proteases and metalloproteinases, leading to widespread consumption of clotting factors and a paradoxical state of both bleeding and thrombosis [4]. VICC leads to bleeding due to consumption of clotting factors by procoagulant venom components. In some patients, endothelial injury and platelet activation also trigger thrombotic microangiopathy (TMA), leading to microvascular thrombosis [10]. Thus, snakebite can paradoxically cause both bleeding and clotting through distinct but overlapping mechanisms. A large case series from Sri Lanka involving 336 patients with confirmed Russell’s viper envenomation reported neurological involvement in approximately 78% of cases [11]. Ptosis and external ophthalmoplegia were among the most common findings, suggestive of presynaptic neuromuscular blockade. Fewer patients experienced classical neuroparalytic syndromes. Follow-up neurophysiological studies confirmed these observations, supporting the neurotoxic effects of viper venom [12].

Our patient presented with respiratory distress, a common feature in severe envenomation. In Russell’s viper bites, this may result from pulmonary edema, neurotoxic respiratory muscle paralysis, or severe metabolic derangements. In this case, the chest radiograph and arterial blood gas analysis were unremarkable, and there was no evidence of pulmonary pathology. Hence, respiratory muscle paralysis is the most plausible explanation for his initial respiratory compromise.

The pathophysiology of cerebral infarction following snakebite is multifactorial and not yet fully understood. Potential mechanisms include venom-induced procoagulant effects causing microthrombi, complement-mediated vasculitis, endothelial injury, cardiotoxic effects leading to arrhythmias and embolism, and hypovolemia-induced hyperviscosity [13]. Phospholipase A2 and other toxic venom components may further contribute to vascular dysfunction. In our case, the absence of typical stroke risk factors, such as dyslipidemia, smoking, arrhythmias, or hypotension, along with the presence of multi-territorial infarcts, strongly suggests a systemic toxin-mediated hypercoagulable state.

Investigations, including echocardiography, carotid and vertebral Doppler studies, and coagulation tests, ruled out conventional sources of embolism. The patient’s lipid profile and homocysteine levels were normal, and imaging revealed no evidence of watershed infarcts or vasculitis. Notably, infarctions occurred rapidly within 24-48 hours of envenomation, supporting an acute and direct toxic effect. Given these findings, the most plausible mechanism is diffuse thrombotic microangiopathy induced by procoagulant venom components.

There is emerging clinical evidence to support this hypothesis. Narang et al. reported a case of middle cerebral artery infarction following Russell’s viper bite [6]. Similarly, Subasinghe et al. described bilateral blindness due to cerebral infarction after envenomation [7], and Murthy et al. documented another case of cerebral infarction linked to viper bite [8]. These cases, like ours, lacked conventional cardiovascular risk factors and presented with rapid-onset stroke symptoms after envenomation.

In summary, this case highlights a rare but serious complication of viper bite-multi-territorial acute ischemic stroke. It reinforces the importance of early neuroimaging in patients with altered sensorium following envenomation and underscores the need to consider procoagulant toxicity as a potential cause of stroke, even in the absence of traditional risk factors.

## Conclusions

This report underscores a rare but severe complication of ischemic stroke following Russell's viper bite, attributed to the procoagulant effects of the venom. Although viper bites are primarily associated with hemorrhagic complications, ischemic neurological issues are also relatively common. Therefore, it is crucial to vigilantly monitor for these complications when treating viper envenomation.
